# Atomic structures of self-assembled epitaxially grown GdSi_2_ nanowires on Si(001) by STM

**DOI:** 10.1038/s41598-018-37015-6

**Published:** 2019-02-04

**Authors:** Sun Kyu Song, Tae-Hwan Kim, Han Woong Yeom

**Affiliations:** 1 Center for Artificial Low Dimensional Electronic Systems, Institute for Basic Science (IBS), Pohang, 37673 Republic of Korea; 2 Department of Physics, Pohang University of Science and Technology (POSTECH), Pohang, 37673 Republic of Korea

## Abstract

Self-assembled rare-earth (RE) silicide nanowires (NWs) on semiconductor surfaces are considered as good candidates for creating and investigating one-dimensional electron systems because of their exceptionally anisotropic growth behavior and metallic property. While detailed atomic structures are essential to understand electronic properties of these NWs, there have been only few successful observations of atomic structures with microscopy and reliable structure models are lacking. Here, we reinvestigate gadolinium silicide NWs with high resolution scanning tunneling microscopy (STM). We observe several different structures of Gd silicide NWs depending systematically on their widths, which consist of two distinct structural elements along the wires. The structure of a wide wire can be understood from that of a two dimensional silicide. Based on these STM observations, we propose new structure models of Gd silicide NWs.

## Introduction

The extreme downscaling of integrated circuit dimensions makes great demands on alternative fabrication approaches in the scale of few nanometers. Nanowires (NWs), structures that have a thickness or diameter limited to tens of nanometers or less and an unconstrained length, have attracted great interest as potential building blocks for alternative nanoscale devices^1–3^. On the other hands, metallic NWs are important model systems to realize a one-dimensional (1D) electron system, which would exhibit various intriguing physical properties, such as broken-symmetry ground states^4–6^, their unconventional topological excitations^7^, and the non-Fermi liquid behavior^8–10^.

Rare-earth (RE) silicide NWs self-assembled on silicon surfaces have been considered as such 1D electron systems due to their highly anisotropic growth features and relatively robust atomic and electronic structures^11–16^. A variety of phases of RE silicide NW systems and the coverage dependent growth behavior have been reported extensively in previous studies^11,17–20^. Their uni-directional growth with an extremely high aspect ratio results from a huge anisotropy in the lattice mismatch between the RE silicide and the Si(001) substrate^11^. Indeed, quite a few exotic 1D phenomena were reported, such as the charge-order fluctuation and the Anderson localization in Y silicide NWs^15^ and the robust metallicity in Gd silicide NWs^14,16^. Although the detailed characterization of atomic structures is crucial to understand any unusual properties of silicide NWs, the direct experimental investigation of their atomic structures has been very limited^12,13,20–22^. Furthermore, satisfactory atomic models explaining experimental findings comprehensively are not available yet.

Among RE silicide NWs, Gd silicide NWs have been intensively studied due to their interesting properties such as the robust metallicity^13,14,16^ and an extremely low Schottky barrier height^23^. Gd silicide NWs exhibit the coexistence of isolated single wires and NW bundles^13,16^. Independent experimental works revealed two different atomic structures of Gd silicide NWs, *c*(2 × 2)^13^ and 5 × 1^16^ structures. No atomic structure model is available for these structures and the relationship between them is not clear.

In this work, we reinvestigate the atomic structure of Gd silicide NWs with high resolution scanning tunneling microscopy (STM). We identify a variety of atomic structures for the Gd NWs, which depend systematically on their widths. Narrow wires exhibit a simple double row ×1 structure. Wider NWs show atomic structures bridging between narrow ones and two-dimentional (2D) silicide islands. Based on our experimental findings, we propose new atomic models explaining the whole structures observed consistently^13,16^.

## Results and Discussions

**Figure 1:**
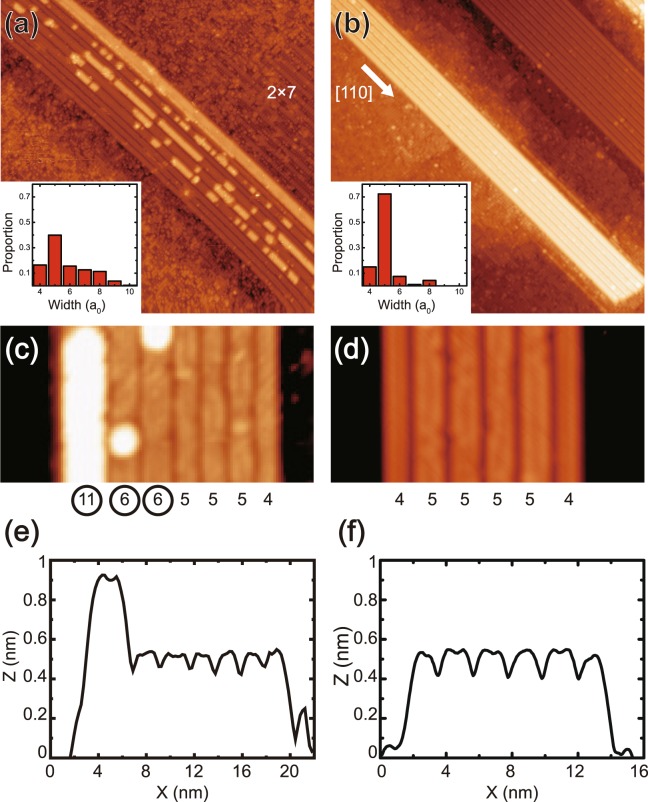
STM images (100 nm × 100 nm) of the two different Gd silicide NWs bundles with different coverages of (**a**) 0.4 ML (V_*S*_ = −1 V) and (**b**) 0.8 ML (V_*S*_ = +1 V). Insets of (**a**) and (**b**) show the statistical distributions of the NW widths in several NW bundles for each coverage. The data were collected from more than 340 of NWs in bundles for each coverage. (**c**,**d**) Close up STM images of the typical NW bundles at (**c**) low and (**d**) high Gd coverage. The numbers below the NWs indicate the corresponding NW width and the circles indicate the NWs with the second layer. (**e**,**f**) STM Line profiles of the NW bundles of (**c**) and (**d**), respectively.

Figure 1(a,b) show the STM images of a Gd silicide NW bundle grown on Si(100) with different coverages of 0.4 and 0.8 ML, respectively. High aspect ratio NWs grow anisotropically with their long axis perpendicular to Si dimer rows of the clean Si(100) surface^22,24^. The growth at different coverages exhibits clear differences as shown in these figures. In contrast to the NW bundle at high coverage [Fig. 1(b)], additional layers appear on the NW bundle at low coverage [Fig. 1(a)]. Furthermore, at low coverage, a partly-ordered 2 × 7 wetting layer appears in large areas as shown in the right side of the NW bundle [Fig. 1(a)]. On the other hand, at high coverage, the 2 × 7 structure appears only in very restricted areas with most parts of the wetting layer disordered as shown in Fig. 1(b). In the LEED study (data not shown here), clear ×7 spots appear at low coverage, but only blurry ×4 spots appear at high coverage, which are consistent with previous LEED works^14,17^ and our STM results.

The distinct growth products are more clearly observed in the close-up STM images of the typical NW bundles at low and high coverage as shown in Fig. 1(c,d), respectively. We determine NW widths by measuring distances between neighboring trenches (rows of dark STM contrast). At low coverage, the NWs in the bundle have various different widths and additional layers appear on wider parts of them. STM line profiles [Fig. 1(e,f)] clearly show that the flat NW bundles and the additional layers on top of them are commonly ∼0.4 nm high, suggesting that the additional layers are the second silicide NW layers. Contrastingly, at high coverage, most of the NWs in the bundle seems to have a regular width (5*a*_0_, where *a*_0_ is the Si lattice constant, 0.384 nm) without any additional layers. This distinct growth behavior depending on the Gd coverage is further supported by the statistical distributions of the NW widths as shown in the insets of Fig. 1(a,b). At both coverages, no NW narrower than 4*a*_0_ is observed in the NW bundle. Although the most dominant width of the wire is 5*a*_0_ at both coverages, the proportions of the 5*a*_0_ NWs are significantly different; 40% at low coverage, but 70% at high coverage. At low coverage, wider NWs than 5*a*_0_ appears with a significantly higher population (∼45% in total) than those at high coverage (∼15%). It can be speculated that the wider NWs are stabilized at the Gd poor growth condition. On the other hand, the 4*a*_0_ NWs are observed with a similar population at both coverages, about 15%. This could be related to the fact that 4*a*_0_ NWs exist only on the edge of a NW bundle [Fig. 1(c,d)]. It is noteworthy that the additional Gd silicide layers are found only on NWs wider than 5*a*_0_. The relationship between the additional layers and the NW widths explains why the additional layers appear only in the low coverage sample. This contrasting growth of overlayers on NWs suggests that the atomic structures of the NWs with various widths may be dissimilar. These observations can largely be explained by the detailed atomic structures of NWs as revealed below.

**Figure 2:**
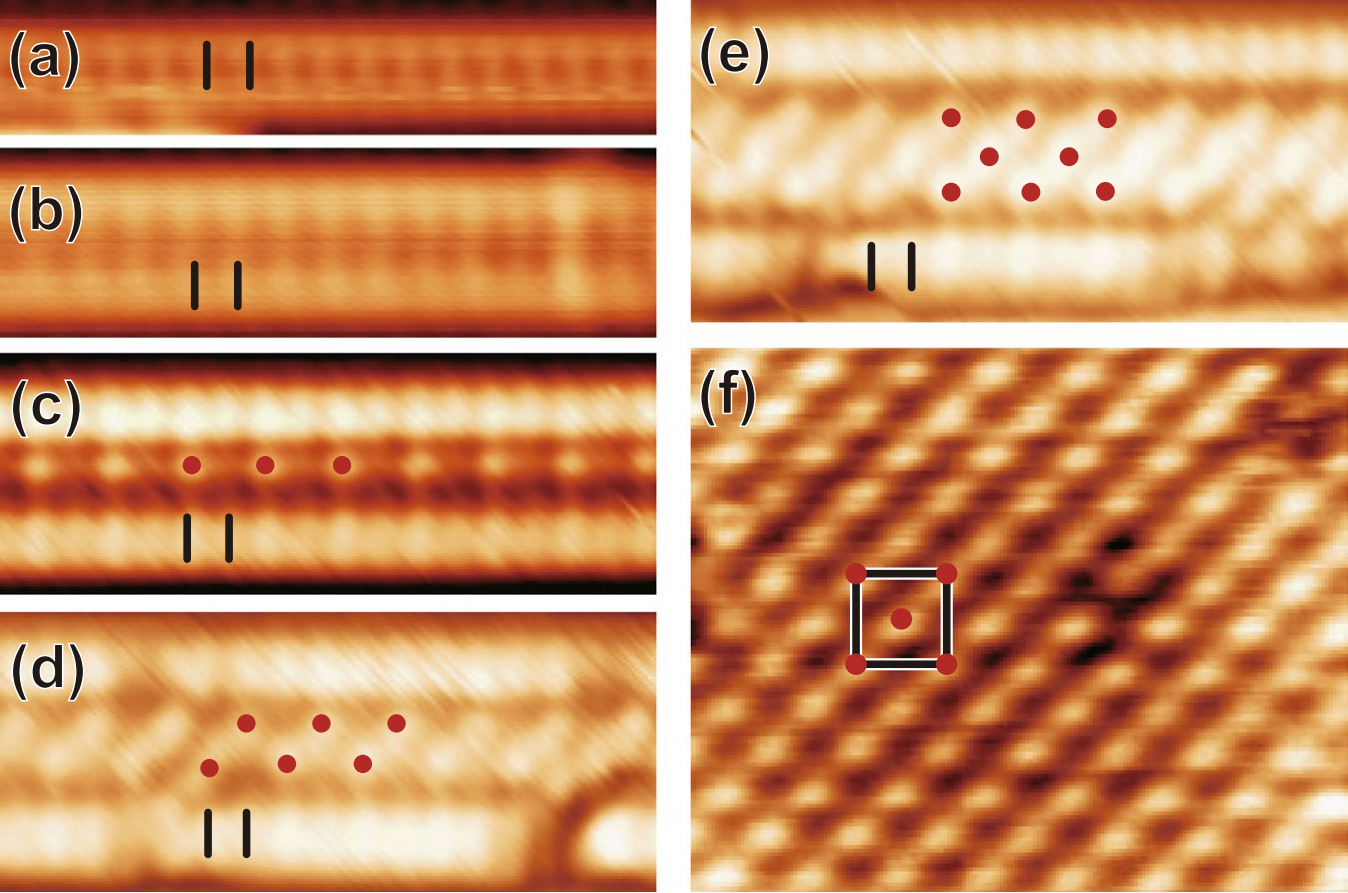
Atom resolved STM images of the NWs of different widths, (**a**) 4*a*_0_, (**b**) 5*a*_0_, (**c**) 6*a*_0_, (**d**) 7*a*_0_ and (**e**) 8*a*_0_ NW and (**f**) the silicide island. The images were measured at 0.1 nA, −1 V in (**a**,**b** and **f**) and 0.1 nA, +1 V in (**c**–**e**). Black bars on the edge of the NWs and red dots on the center of the NWs in (**c**–**e**) show the periodicities along the edge and the center of the NWs, respectively. The black box with red dots in (**f**) shows the c(2 × 2) structure on the silicide island.

For such a purpose, we have performed high-resolution STM on NWs with various widths. Atomically resolved STM images in Fig. 2 clearly reveal a variety of atomic structures of NWs with different widths from 4*a*_0_ to 8*a*_0_. The 4*a*_0_ and the 5*a*_0_ wide NWs have commonly two rows of protrusions with a very simple × (1*a*_0_) periodicity along the wire as shown in Fig. 2(a,b). The only difference is the spacing between the two rows. However, wider NWs than 5*a*_0_ have distinct structures on the edge and the center of the wire. While a similar ×1 structure appears along the edge of the wires, distinct spotty rows of protrusions with a ×2 periodicity appear along the center of the NWs between the edge rows [Fig. 2(c–e)]. For 6*a*_0_, 7*a*_0_, and 8*a*_0_ NWs or wider ones, one, two, and three or more rows with a period of 2*a*_0_ are added systematically in the center of NWs in a staggered way. These extra rows of protrusions form eventually a *c*(2 × 2) structure on a 8*a*_0_ (or wider) NWs as shown in Fig. 2(c,e). The *c*(2 × 2) structure on the NW center is apparently very similar to the *c*(2 × 2) structure observed on 2D Gd silicide islands formed at a higher coverage [Fig. 2(f)]. As a result, we can categorize Gd NWs in NW bundles into two groups: 4*a*_0_ or 5*a*_0_ NWs and wider ones based on their distinct structures. These are the major findings of the present work. The previous observation of the NWs with a *c*(2 × 2) structure is consistent with the present observation for wide wires. This work seems to neglect the narrow wires^13^. The other report of the wires with a 5 × 1 structure must be related to the present observation of the 5*a*_0_ wide wires with a ×1 periodicity along the wire^16^. That is, the former and the latter works seem to have focused on low and high coverage (Gd-poor and -rich) growth, respectively.

**Figure 3:**
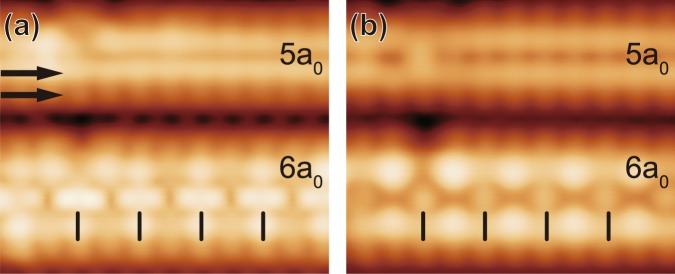
Bias dependent STM images of the 5*a*_0_ NW and the 6*a*_0_ NW for (**a**) filled states (V_*S*_ = −1 V) and (**b**) empty states (V_*S*_ = +1 V). Black bars on the inner edge of the 6*a*_0_ NW indicate the periodicity along the inner edge of the 6*a*_0_ NW. Arrows on 5*a*_0_ NW indicate two rows of protrusions.

In order to gain further insight into the NW structures with different widths, we conduct bias-dependent STM measurements. Figure 3(a,b) show the high-resolution STM images in filled and empty states at 78 K, respectively. They reveal that each edge of NWs is formed with two ×1 rows of protrusions; one row (closer to the center) has a brighter contrast than other in both filled and empty states. This suggests that the inner edge rows are higher then the outer ones. Moreover, the inner edge rows of a 6*a*_0_ NW show a clear bias dependent corrugation. The periodicity along the edge of a 6*a*_0_ NW becomes doubled at 78 K, and the phase of the STM modulation along the inner edge changes by 180° when the bias polarity is reversed, that is, the up and down is reversed. Making the structure more complex, the 2*a*_0_ central row of a 6*a*_0_ NW shows dimer-like elongated or split structures in filled states, which become single spotty protrusions in empty states. The center of a 6*a*_0_ NW looks darker than the inner edge rows in empty states, while the apparent height of the center row is almost the same as that of the inner edges in filled states. Basically, wider NWs than 5*a*_0_ features 2*a*_0_ structures along both edges and central parts with strong bias dependence or electronic effects. At present, judged from the similarity of the STM images, we think that the (inner and outer) edge rows of narrow and wide NW have a similar structure while those of wider ones have an extra 2*a*_0_ modulation. The 2*a*_0_ modulation along edge rows could be triggered by the ×2 [*c*(2 × 2)] structure of the central part in our best guess.

Before constructing a reasonable atomic structure model, it is helpful to compare our results with the previous STM studies. To our best knowledge, two different structures of Gd silicide NWs were proposed; the *c*(2 × 2) structure similar to the bulk silicide surface structure^13^, and the ×1 structure model based on the 5*a*_0_ NW consisting of two Si dimer rows with a ×1 periodicity^16^. At first glance, the appearance of the ×1 periodicity on a 5*a*_0_ NW seems consistent with this 5*a*_0_ NW model. However, NWs wider than 5*a*_0_ have both ×1 and ×2 structures on the edge and the center of the wires, respectively, requesting an extra structural element. On the other hand, the *c*(2 × 2) structure appears only where the width is wider than 7*a*_0_ and only along the central part of NWs. From this comparison, we can confirm that there is no satisfactory atomic model consistent with our observation of the systematic change of the NW structure depending upon their width.

**Figure 4:**
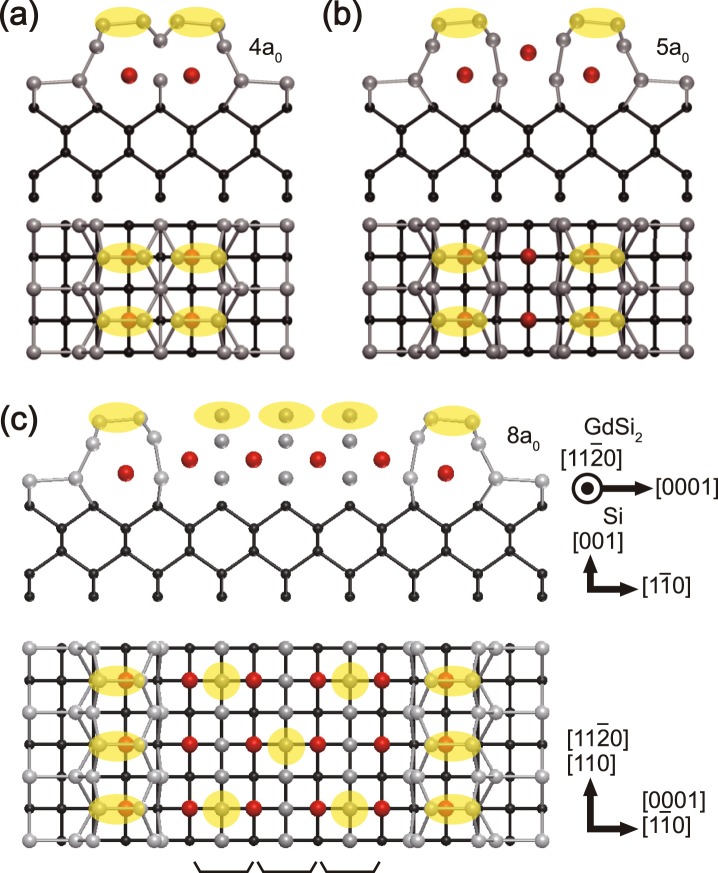
Cross-sectional side view and top view of the atomic structure models of (**a**) 4*a*_0_, (**b**) 5*a*_0_, and (**c**) 8*a*_0_ NW. Silicon atoms in silicide are large gray and bulk silicon atoms are smaller black circles, and Gd atoms are large red circles. The translucent yellow circles indicate the bright positions of the STM images. The black bars below the wire in (**c**) indicate the identical units of the top-most structure of the center of the wider NWs than 5*a*_0_ as the width of the wire increases by 1*a*_0_.

Recently, YSi_2_ NWs was also reported to exhibit discrete widths varying between 4*a*_0_ and 8*a*_0_^20^ (The definition of NW width in ref.^20^ differs by 2*a*_0_ from our own definition.). The majority of YSi_2_ NWs are 4*a*_0_ and 5*a*_0_ in width. This observation is consistent with what are observed in the Gd NWs at high coverage [Fig. 1(b,d)]. Besides, YSi_2_ NWs wider than 5*a*_0_ show the periodicity doubling along the wire direction, which is qualitatively consistent with our experimental observation. Since bulk Gd silicide has a similar atomic structure to that of YSi_2_, it is worth considering the atomic structures proposed for YSi_2_ NWs. The YSi_2_ structure models^20^ are considered in accord with a 5*a*_0_ NW^15^, whose STM images are basically the same as those of 5*a*_0_ Gd silicide NW^16^. This model consists of two Si dimer rows with a ×1 periodicity and three rows of metal atoms as shown in Fig. 4(b). For the 5*a*_0_ NWs, the proposed atomic structure model can explain the STM images well and can easily lead to a similar structure for a 4*a*_0_ NW by removing a central metal row [Fig. 4(a)]. However, the structure models for NWs wider than 5*a*_0_ was also constructed as an extension of this Si dimer row structure, basically for 7*a*_0_, 9*a*_0_, and so on^20^. These authors assumed yttrium deficiency to explain the *c*(2 × 2) pattern in wider NWs. The expected STM images for this model (and a similar one for a 6*a*_0_ NW) are not consistent at all with the present case of the Gd silicide NWs [Fig. 3] due mainly to the presence of the Si dimer rows in the model along the center of a NW. In addition, since the model is composed of Si dimer rows separated by metal atomic rows, any stable NW structure with a width of 6*a*_0_, 8*a*_0_ and so on are not easily possible. Note also that clear atomic resolution STM images for NWs with various width were not available in those previous studies, which would have presented a critical test of structure models. Therefore, a different approach to structure model is obviously needed.

In order to construct the structure of NWs with different widths, we start with the previous ×1 structure model of a 5*a*_0_ NWs^16,20^. It has two GdSi_2_ edge units in the hexagonal-GdSi_2_ (*h*-GdSi_2_) structure^18^ and Gd atoms between them, as shown in Fig. 4(b). The edge *h*-GdSi_2_ row has Si dimers on top. Since a 4*a*_0_ NW shows the same ×1 periodicity but no spacing between the dimers, a 4*a*_0_ NW would have similar two *h*-GdSi_2_ edge units but no Gd rows in between as shown in Fig. 4(a). This model is consistent with the present STM images. We however have to consider the buckling of Si dimers as shown in Fig. 4 in order to explain the different height of the inner and outer rows mentioned above for the images in Fig. 3. For wider NWs than 5*a*_0_, we need to consider the periodicity and the bias dependent features of STM images. The ×1 periodicity and the bias dependent features of the edges of wider NW in Fig. 3 are consistent with those of 4*a*_0_ and 5*a*_0_ NWs. This suggests that the structure of the NW edges would be similar to the structure of the 4*a*_0_ or 5*a*_0_ NWs as mentioned above.

In contrast, a distinct *c*(2 × 2) structure appears along the center of a 8*a*_0_ NW and this structural element seems consistent also in 6*a*_0_ and 7*a*_0_ NWs. The *c*(2 × 2) structure on the NW center is obviously similar to the *c*(2 × 2) structure observed on 2D Gd silicide islands (films) and the surface of bulk silicides, which is known to have the orthorhombic-GdSi_2_ (*o*-GdSi_2_) structure^19^ [Fig. 2(f)]. This is also suggested by the growth of an extra silicide layer only on a wider NW than 5*a*_0_. It is natural to think that a sufficiently wide NW has a 2D silicide structure except for its edges. Thus, we need to consider the atomic structures of the edge and the center separately for the wider NWs than 5*a*_0_. Starting from the above ideas, we propose a kind of a hybrid structure model of the wider (8*a*_0_) Gd silicide NW as shown in Fig. 4(c). The wider NWs are assumed to have the same *h*-GdSi_2_ edge structure as the 5*a*_0_ case while the center of the NWs is composed of the *o*-GdSi_2_ island structure. Since these crystal structures of the Gd silicide could be represented by stacking the structural unit of the Gd silicide^18^, the *h*-GdSi_2_ is stacked with consistent shifting perpendicular to *c* axis (ABAB… stacking), and the *o*-GdSi_2_ is stacked alternatively along *a* and *b* axes (ABCDABCD… stacking). However, in the limit of very thin films with only one structural unit height the distinction between these two structures is not obvious and can be connected laterally as shown in Fig. 4(c).

Unfortunately, at present a detailed atomic structure of the *c*(2 × 2) surface reconstruction of *o*-GdSi_2_ is not known and thus the details of the *c*(2 × 2) structure in the present NW model is also unclear. In order to have a similar height for the central and edge rows as shown in the present STM images [Figs 2 and 3], we stack an extra Si layer in the model as shown in Fig. 4(c). We think that this extra Si layer would provide a structural degree of freedom for the *c*(2 × 2) reconstruction and also makes the wide NWs favored for a Gd poor growth condition. The structure models for 6*a*_0_ and 7*a*_0_ NWs are straightforward from this model with reduced atomic rows of the *o*-GdSi_2_ layer. The abrupt junctions between two NWs having different widths are observed previously^15,20,25^, which indicate that the structures of the center of wider NWs (6*a*_0_, 7*a*_0_, 8*a*_0_ and go on) are formed with a consistent structure. A further experimental and theoretical work for the *c*(2 × 2) surface reconstruction of *o*-GdSi_2_ can solve the major open question within the present structure model.

## Conclusions

Self-assembled Gd silicide NWs are grown on a clean Si(100) surface by depositing Gd atoms and high-resolution STM images reveal their atomic structures with unprecedented resolution and details. We find that different Gd coverages lead to different NW structures with discrete width distributions. STM images reveal distinct atomic structures of narrow (4*a*_0_ and 5*a*_0_ wide) NWs and wider ones clearly. The narrow NWs have double buckled dimer rows with a periodicity of *a*_0_ along the wire. The wider wires has extra central row structures with a 2*a*_0_ periodicity, which systematically evolves to a *c*(2 × 2) structure observed in the 2D silicide islands of orthorhombic-GdSi_2_. The structure models for the narrow wires are adopted from the model previously proposed with double Si dimer rows in parallel. The models for the wider NWs are newly proposed which hybrid the edges with Si dimer rows and the 2D-like orthorhombic-GdSi_2_ rows with a *c*(2 × 2) reconstruction between edges. The details of the *c*(2 × 2) reconstruction remains to be determined. This work would provide an important basis for the study of intriguing electronic properties found in various self-assembled rare earth silicide NWs.

## Methods

Our experiments were performed in two different ultrahigh vacuum chambers equipped respectively with a commercial variable temperature STM (Omicron, Germany) operated at room temperature and a commercial cryogenic STM (SPECS, Germany) at 78 K. An *n*-type Si(100) substrate was cleaned by flash heating to 1450 K a few times. The clean double domain 2 × 1 surface was confirmed with low energy electron diffraction (LEED) and STM. Gd atoms were deposited onto the clean surface by using an electron-beam evaporator. The substrate was kept at temperatures of 850-900 K while depositing Gd atoms. After depositions, the substrate was cooled down rapidly to room temperature^16,24^. Gd coverages [1 ML = Si(001) surface atomic density of 6.8 × 10^14^ atoms/cm^2^] were controlled by changing deposition times with a fixed deposition rate and determined by LEED patterns^14,17^ and large-scale STM images.
